# Otimização da Terapia de Reperfusão no Infarto Agudo do Miocárdio com Supradesnível do Segmento ST por Meio de Telemedicina Baseada no WhatsApp^®^

**DOI:** 10.36660/abc.20201243

**Published:** 2022-01-11

**Authors:** Alessandra Batista Teixeira, Leonardo Fiaschi Zancaner, Fernando Fonseca de França Ribeiro, José Paulo Pintyá, André Schmidt, Benedito Carlos Maciel, José Antônio Marin, Carlos Henrique Miranda

**Affiliations:** 1 Universidade de São Paulo Faculdade de Medicina de Ribeirão Preto Divisão de Medicina de Emergência do Departamento de Clínica Médica Ribeirão Preto SP Brasil Universidade de São Paulo Faculdade de Medicina de Ribeirão Preto - Divisão de Medicina de Emergência do Departamento de Clínica Médica,Ribeirão Preto, SP - Brasil; 2 Universidade de São Paulo Faculdade de Medicina de Ribeirão Preto Centro de Cardiologia Ribeirão Preto SP Brasil Universidade de São Paulo Faculdade de Medicina de Ribeirão Preto - Centro de Cardiologia,Ribeirão Preto, SP - Brasil

**Keywords:** Infarto do Miocárdio com Supra Desnível do Segmento ST (IAMCSST, Síndrome Coronariana Aguda, Telemedicina/tendências, Reperfusão/terapia

## Abstract

**Fundamento:**

Cerca de 40% dos pacientes com infarto agudo do miocárdio com supradesnível do segmento ST (IAMCSST) no Brasil não recebem terapia de reperfusão.

**Objetivo:**

A utilização de uma rede de telemedicina baseada no WhatsApp^®^ poderia aumentar a porcentagem de pacientes que recebem terapia de reperfusão.

**Métodos:**

Estudo transversal do tipo antes e depois da organização de uma rede de telemedicina para envio e análise do eletrocardiograma através do WhatsApp® dos pacientes suspeitos de IAMCSST oriundos dos 25 municípios integrantes do Departamento Regional de Saúde de Ribeirão Preto (DRS−XIII), para hospital terciário que poderia autorizar a transferência imediata do paciente utilizando o mesmo sistema. O desfechos analisados foram a porcentagem de pacientes que receberam terapia de reperfusão e a taxa de mortalidade intra-hospitalar. Considerou-se valor de p <0,05 como estatisticamente significativo.

**Resultados:**

Foram comparados 82 pacientes antes desta rede (1º de fevereiro de 2016 a 31 de janeiro de 2018) com 196 pacientes depois da implantação da mesma (1º de fevereiro de 2018 a 31 de janeiro de 2020). Após a implantação da rede, houve aumento significativo da proporção de pacientes que receberam terapia de reperfusão (60% vs. 92%), risco relativo (RR): 1,594 [intervalo de confiança (IC) 95% 1,331 – 1,909], p <0,0001 e redução da mortalidade intra-hospitalar (13,4% vs. 5,6%), RR: 0,418 [IC 95% 0,189 – 0,927], p = 0,028.

**Conclusão:**

Rede de telemedicina baseada no WhatsApp^®^ associou-se a aumento da porcentagem de pacientes com IAMCSST que receberam terapia de reperfusão e a redução na mortalidade intra-hospitalar.

## Introdução

As doenças cardiovasculares são a principal causa de mortalidade mundial, inclusive no Brasil.^1^ O infarto agudo do miocárdio com supradesnível do segmento ST (IAMCSST) é o responsável por grande parte dos eventos fatais dessa etiologia. De acordo com divulgação pelo DATASUS, houve 142.982 hospitalizações por IAM no ano de 2018 no Brasil.^1^ Nas últimas décadas, houve importante redução na morbimortalidade do IAMCSST, principalmente com o desenvolvimento das terapias de reperfusão (fibrinolíticos e angioplastia primária).^2^ Contudo, para obtenção deste benefício, é necessário o reconhecimento precoce deste evento coronariano, o qual usualmente se baseia na anamnese e no eletrocardiograma (ECG) de urgência, de modo a possibilitar a organização do rápido direcionamento desses pacientes para centros terciários preparados para oferecer tais modalidades de terapia.

Registro brasileiro de síndromes coronarianas agudas evidenciou que apenas 61,2% dos pacientes com IAMCSST receberam alguma terapia de reperfusão para o tratamento (35,9% recebendo angioplastia primária e 25,3% recebendo terapia fibrinolítica).^3^ Ou seja, uma grande porcentagem de pacientes em nosso país, principalmente aqueles oriundos de serviços públicos, ainda não recebe terapia de reperfusão em tempo hábil, fato que impactará diretamente na sobrevida e no comprometimento funcional do ventrículo esquerdo desses pacientes e na consequente insuficiência cardíaca de muitos casos de IAMCSST.

O objetivo dessa investigação foi avaliar se a implantação de uma rede de telemedicina para envio do ECG de pacientes com suspeita de supradesnível do segmento ST, utilizando-se de uma plataforma simples de comunicação digital (WhatsApp^®^) para análise imediata em um centro terciário, ocasionaria aumento na porcentagem de pacientes que recebem a terapia de reperfusão dentro das 12 horas iniciais do IAMCSST. Adicionalmente, avaliou se o impacto da organização do fluxo de liberação imediata para transferência hospitalar do paciente suspeito de IAMCSST utilizando-se deste mesmo recurso de comunicação na mortalidade intra-hospitalar acarretada por esse evento coronariano.

## Métodos

Estudo transversal do tipo antes e depois, no qual se comparou a porcentagem de pacientes que receberam a terapia de reperfusão dentro do período de 12 horas para o tratamento do IAMCSST, antes e depois da implantação de uma rede (Rede Supra) para envio e análise à distância do ECG em suspeitos dessa patologia utilizando-se de uma plataforma acessível de comunicação digital (WhatsApp^®^) para um centro terciário de cardiologia.

**Figura 1:**
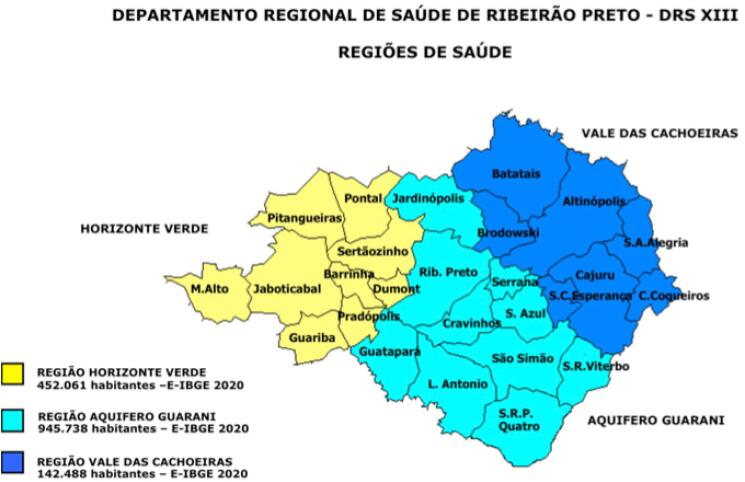
– Mapa geográfico dos municípios constituintes do Departamento Regional de Saúde de Ribeirão Preto (DRS–XIII); assim como sua subdivisão nas regiões do Horizonte Verde, Aquífero Guarani e Vale das Cachoeiras.

Esta rede englobou os 25 municípios integrantes do Departamento Regional de Saúde de Ribeirão Preto (DRS-XIII). Nessa fase inicial, optou-se por não incluir o município de Ribeirão Preto na coleta de dados, pois, devido à sua localização, este município já apresentava maior facilidade inerente de encaminhamento desses pacientes para um hospital terciário. A Figura 1 mostra os municípios integrantes da DRS-XIII, assim como a sua subdivisão em três regiões denominadas: Horizonte Verde, Aquífero Guarani e Vale das Cachoeiras. A central de recebimento desses ECG estava localizada na Unidade Coronariana da Unidade de Emergência do Hospital das Clínicas da Faculdade de Medicina de Ribeirão Preto da Universidade de São Paulo (HC-FMRP/USP), também localizada no município de Ribeirão Preto, e este é um hospital terciário de referência para o atendimento e tratamento exclusivo de emergências para toda essa região.

Este estudo foi aprovado pelo Comitê de Ética em Pesquisa de nosso hospital e seguiu as recomendações da declaração de Helsinki.

### Implantação da rede supra

Foi realizada uma consulta inicial junto ao Conselho Regional de Medicina, o qual foi favorável à utilização do WhatsApp^®^ para envio do ECG entre médicos. O projeto foi apresentado e aprovado na Comissão Regional Intergestores da DRS-XIII junto aos secretários de saúde dos municípios constituintes.

**Figura 2:**
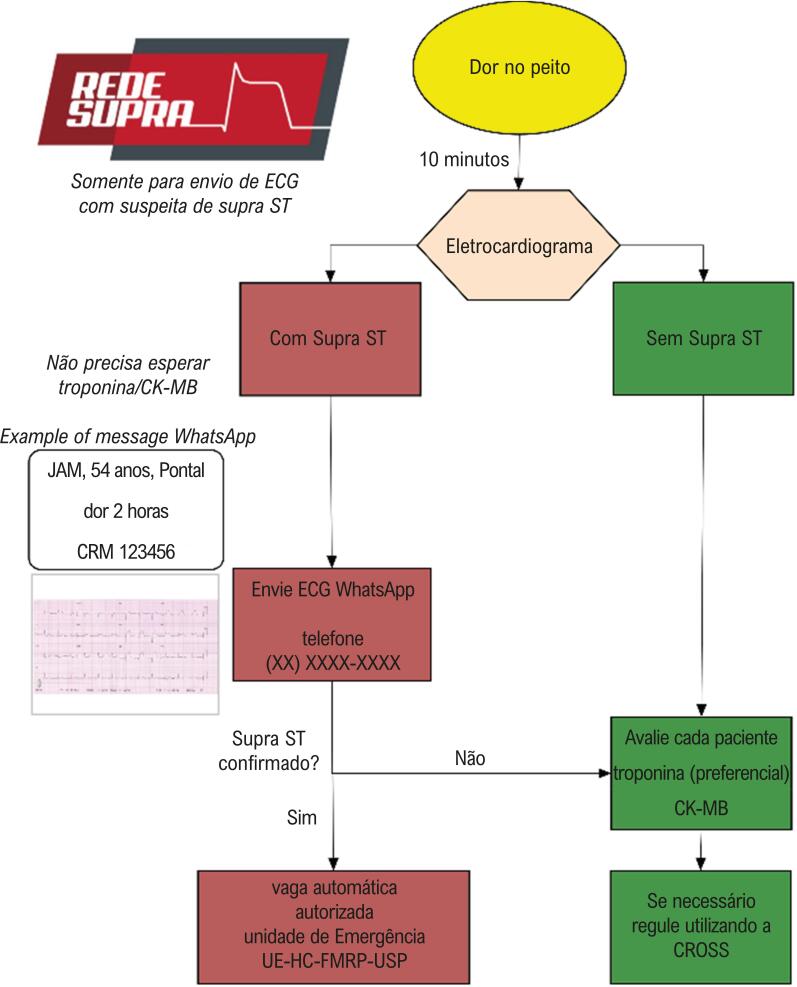
– Fluxograma de orientação do funcionamento de rede de telemedicina baseada no WhatsApp^®^ para envio de eletrocardiograma (ECG) suspeito de infarto agudo do miocárdio com supradesnível do segmento ST (IAMCSST) e organização do fluxo de transferência deste paciente para o hospital terciário. CRM: Conselho Regional de Medicina; UE-HC-FMRP: Unidade de Emergência do Hospital das Clínicas da Faculdade de Medicina de Ribeirão Preto; CROSS: Central de Regulação de Ofertas de Serviços de Saúde da Secretaria de Saúde do Estado de São Paulo.

Dois treinamentos destinados aos enfermeiros responsáveis pela unidade de pronto-atendimento de cada município foram realizados para demostrar a importância do diagnóstico precoce e encaminhamento rápido do paciente com IAMCSST para um hospital terciário, visto que nenhum desses munícipios dispunha de serviço de angioplastia primária, e medicamentos fibrinolíticos estavam disponíveis somente em quatro deles. Foi realizado treinamento prático de como realizar ECG de qualidade que permita o diagnóstico correto e um questionário sobre os recursos disponíveis em cada unidade para o atendimento desses pacientes. Após essas duas atividades, o fluxograma detalhado na Figura 2 foi apresentado aos médicos coordenadores dessas unidades, contendo as orientações sobre o envio do ECG via WhatsApp^®^ na suspeita de IAMCSST e a organização do fluxo para transferência desses pacientes para o hospital de referência (ver Figura 2).

Disponibilizou-se, ainda, um telefone celular exclusivo para este tipo de comunicação. Os cardiologistas assistentes da unidade coronariana da unidade de emergência do HC-FMRP/USP ficaram responsáveis pela interpretação desses ECG. Foram criadas duas respostas-padrão: IAMCSST confirmado, autorizada a transferência imediata para unidade de emergência, e IAMCSST não confirmado, se necessário regular via CROSS (Central de Regulação de Ofertas de Serviços de Saúde). No dia 1º de fevereiro de 2018, o número do celular mencionado foi disponibilizado para todos esses municípios.

### Coleta de dados

Os dados retrospectivos anteriores à instalação da Rede Supra foram adquiridos por meio de revisão dos prontuários de pacientes hospitalizados em nosso hospital com diagnóstico principal de IAMCSST identificados a partir dos seguintes códigos internacionais de doenças (CID-10): I21.0 (infarto agudo transmural da parede anterior do miocárdio, I21.1 (infarto agudo transmural da parede inferior do miocárdio), I21.2 (infarto agudo transmural do miocárdio de outras localizações), I21.3 (infarto agudo transmural do miocárdio, de localização não especificada), I22.0 (infarto do miocárdio recorrente da parede anterior), I22.1 (infarto do miocárdio recorrente da parede inferior), I22.8 (infarto do miocárdio recorrente de outras localizações), I22.9 (infarto do miocárdio recorrente de localização não especificada). Foram analisados somente os casos encaminhados pelos 25 municípios constituintes da DRS-XIII em período de 2 anos antes da implantação desta rede de telemedicina. Para definição do diagnóstico de IAMCSST, foram utilizados os critérios da quarta definição universal de infarto do miocárdio.^4^

Depois da instalação da Rede Supra, os dados foram levantados prospectivamente por enfermeira da unidade coronariana que revisava semanalmente as mensagens e os ECG enviados por WhatsApp^®^ para essa central e, posteriormente, conferia no prontuário de cada paciente as informações necessárias para este estudo durante o período de 2 anos subsequentes à implantação desta rede.

O desfecho primário avaliado foi a porcentagem de pacientes que receberam alguma terapia de reperfusão (fibrinolítico, angioplastia primária ou espontânea) dentro de 12 horas do início dos sintomas de dor torácica. Os desfechos secundários avaliados foram a taxa de mortalidade intra-hospitalar e o tempo entre o início da dor torácica até o início da terapia de reperfusão. Para os pacientes com reperfusão espontânea ou para aqueles que não receberam nenhuma terapia de reperfusão, considerou-se o tempo entre o início da dor até a admissão hospitalar.

### Análise estatística

Para determinação do tamanho amostral utilizando-se do teste do Qui-quadrado, assumiu-se uma porcentagem de 50% dos pacientes oriundos dessa região que receberia terapia de reperfusão antes da implantação dessa rede, com base em dados históricos recentes de nossa instituição. Além disso, considerou-se que essa porcentagem se elevaria para 80% depois da implantação dessa rede, detectando-se essa diferença com poder de 80% e um nível de significância de 5%. Assim, seria necessária a inclusão de no mínimo 50 pacientes (antes) e 50 pacientes (após) para o teste dessa hipótese.

Utilizou-se o teste de Shapiro-Wilk para a avaliação do tipo de distribuição das variáveis quantitativas. As variáveis quantitativas com distribuição normal foram expressas em média ± desvio-padrão, e as demais variáveis em mediana e intervalo interquartil (IQ). Para comparação entre duas variáveis quantitativas com distribuição normal, utilizou-se do teste t de Student não pareado; e para aquelas com outro tipo de distribuição, utilizou-se o teste de Mann-Whitney. As variáveis qualitativas foram expressas em frequências e porcentagens. Para comparação entre duas ou mais variáveis qualitativas, utilizou-se do teste do Qui-quadrado. Para avaliação da associação entre duas variáveis, calculou-se o risco relativo (RR), assim como o seu intervalo de confiança de 95% (IC 95%). Considerou-se um p-valor bicaudal <0,05 como estatisticamente significativo. A análise estatística e a construção dos gráficos foram realizadas no *software* estatístico GraphPad Prism versão 7.00 (Califórnia, USA).

## Resultados

No período de 1º de fevereiro de 2018 a 31 de janeiro de 2020, foram avaliados os ECG de 1.847 pacientes enviados por meio dessa rede. Suspeitou-se da ocorrência de IAMCSST em 280 (15%) desses exames, o qual foi confirmado em 196 pacientes (11%) após avaliação clínica e repetição do ECG no cenário intra-hospitalar. O tempo entre o recebimento do ECG e o envio da resposta foi inferior a 10 minutos na grande maioria dos casos. As demais características dos pacientes cujos ECG foram analisados por esta rede de telemedicina são mostradas na Tabela 1.

**Tabela 1:** – Caracterização dos pacientes cujos eletrocardiogramas foram enviados pela Rede Supra por meio do WhatsApp® no período de 01 de fevereiro de 2018 a 31 de janeiro de 2020

Característica		n = 1847
ECG com supradesnível ST suspeito, n (%)		280(15)
ECG com supradesnível ST confirmado, n (%)		196(11)
**Tempo de resposta, n (%)**		
<10 min		1.651(89)
10 a 30 min		125(07)
30 a 60 min		36(02)
>60 min		35(02)
**Faixa etária, n (%)**		
<40 anos		268(15)
≤40 a <50 anos		261(14)
≤50 a <60 anos		379(21)
≤60 a <70 anos		395(21)
≥70 anos		416(23)
Não informada		128(06)
**Gênero, n (%)**		
Masculino		1.033(56)
Feminino		541(29)
Não informado		273(15)
**Origem, n (%)**		
Horizonte Verde		645(35)
Aquífero Guarani		596(32)
Não informado		332(18)
Vale das Cachoeiras		274(15)

*ECG: eletrocardiograma; min: minutos.*

As características demográficas e clínicas dos pacientes com diagnóstico de IAMCSST atendidos em nosso serviço antes e depois da implantação desta rede são expostas na Tabela 2. Não foi observada diferença em relação à idade. Apesar de predominância do gênero masculino nos dois períodos analisados, observou-se nítido aumento da proporção do gênero feminino no segundo período. Não foi observada qualquer diferença em relação aos antecedentes pessoais desses pacientes, assim como em relação às paredes do ventrículo esquerdo acometidas.

**Tabela 2:** – Características dos pacientes na admissão hospitalar antes e depois da implantação da Rede Supra

Característica		Antes (n = 82 pacientes)	Depois (n = 196 pacientes)	p-valor
**Demográficas**				
Idade, média ± dp		60 ± 11	61 ± 12	0,676
Gênero masculino, n (%)		65(79)	123(63)	0,007
**Antecedentes pessoais, fatores de risco**				
Hipertensão arterial, n (%)		46(56)	112(57)	0,873
Diabetes, n (%)		21(26)	57(29)	0,661
Dislipidemia, n (%)		21(26)	42(21)	0,448
Tabagismo, n (%)		41(50)	92(47)	0,641
IAM prévio, n (%)		6(07)	21(11)	0,383
Angioplastia prévia, n (%)		2(02)	15(08)	0,098
CRVM prévia, n (%)		0(00)	00(00)	1,000
**Exame físico na admissão**				
PAS (mmHg), média ± dp		135 ± 27	122 ± 27	0,0009
PAD (mmHg), média ± dp		74 ± 15	82 ± 17	<0,0001
FC (batimentos/min), média ± dp		80 ± 20	85 ± 18	0,072
PAS < 90 mmHg, n (%)		7(09)	8(04)	0,134
**Parede VE acometida, n (%)**				0,155
Anterior		27(33)	90(46)	
Inferior		53(65)	96(49)	
Outra		2(02)	10(05)	
Troponina (μg/L), mediana (IQ)		9,87(3,28-20,09)	13,50(5,00-30,00)	0,058
**Origem, n (%)**				0,043
Vale das Cachoeiras		18(22)	68(35)	
Horizonte Verde		29(35)	62(32)	
Aquífero Guarani		35(43)	66(34)	
Duração internação (dias), mediana (IQ)		5(4-9)	5(4-9)	0,845

*dp: desvio padrão; IAM: infarto agudo do miocárdio; CRVM: cirurgia de revascularização miocárdica; PAS: pressão arterial sistólica; PAD: pressão arterial diastólica; FC: frequência cardíaca; VE: ventrículo esquerdo; IQ: intervalo interquartil.*

**Figura 3:**
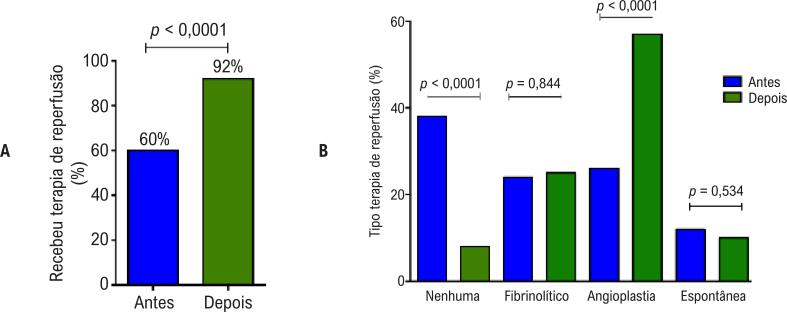
– Gráfico de barras mostrando a porcentagem de pacientes que receberam alguma terapia de reperfusão antes e depois da instalação da Rede Supra (A), assim como os tipos de terapia de reperfusão recebidos para o tratamento do infarto agudo do miocárdio com supradesnível do segmento ST (IAMCSST) (B). Espontânea: recanalização coronária/reperfusão miocárdica espontânea.

Após a implantação da Rede Supra, observou-se um aumento estatisticamente significativo da proporção de pacientes que receberam terapia de reperfusão para o tratamento agudo do IAMCSST, 49/82 (60,00%) *versus* 180/196 (92,00%), RR: 1,594 (IC 95% 1,331 – 1,909), p <0,0001 (Figura 3A). Em relação ao tipo de terapia de reperfusão utilizada no tratamento, observou-se também aumento estatisticamente significativo na proporção de pacientes tratados com angioplastia primária; com manutenção da proporção de pacientes que receberam algum agente fibrinolítico; ou que apresentaram reperfusão espontânea, ou seja, sem trombólise farmacológica ou mecânica (Figura 3B).

**Figura 4:**
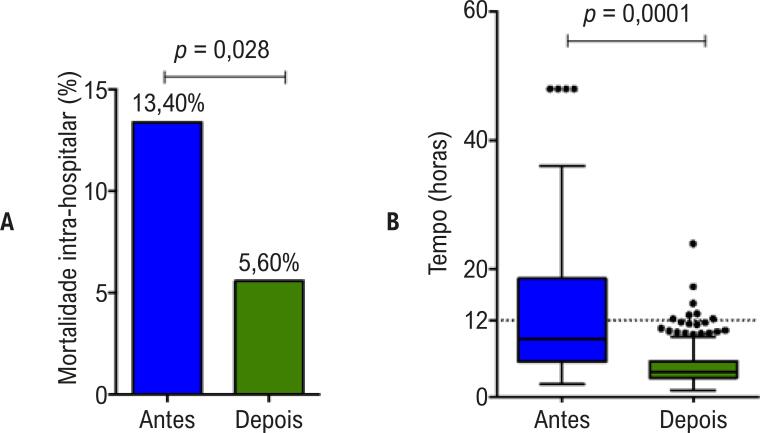
– Gráfico de barras mostrando a mortalidade intra-hospitalar (A) e box-plot mostrando o tempo entre o início dos sintomas e o início da terapia de reperfusão (B) dos pacientes com diagnóstico confirmado de infarto agudo do miocárdio com supradesnível do segmento ST (IAMCSST) antes e depois da instalação da Rede Supra.

Observou-se, ainda, significativa redução na taxa de mortalidade intra-hospitalar dos pacientes com IAMCSST em nosso serviço, 11/82 (13,40%) versus 11/196 (5,60%), RR: 0,418 (IC 95% 0,189 – 0,927), p = 0,028 (Figura 4A). Esses valores indicam um número necessário para tratar (NNT) de 12, correspondente à redução de mortalidade. Em relação ao tempo entre o início da dor torácica e o início da terapia de reperfusão, houve redução estatisticamente significativa de 9 h [IQ 6 – 19] no período anterior, para 4 h [IQ 3 – 6] no período posterior à rede, p <0,0001.

## Discussão

Esta investigação mostrou que a organização de uma rede de telemedicina utilizando plataforma de comunicação digital acessível (WhatsApp^®^) para o envio do ECG, sua análise especializada e a organização do fluxo de pacientes com suspeita de IAMCSST para hospital terciário de referência no tratamento desta patologia associou-se a aumento significativo da proporção de pacientes que receberam terapia de reperfusão, diminuição do tempo para implementação desta terapia e expressiva redução da taxa de mortalidade intra-hospitalar.

Há inúmeras evidências científicas de que o início rápido da terapia de reperfusão para o tratamento do IAMCSST reduz significativamente as complicações, principalmente pela minimização do dano miocárdico.^5,6^ Partindo dessa observação, a organização de redes regionais de atendimento a pacientes com IAMCSST é preconizada na tentativa de se agilizar seu tratamento.^7-9^

Também em países em desenvolvimento, como o Brasil, porcentagem expressiva de pacientes com IAMCSST ainda não recebe terapias de reperfusão dentro de janela temporal adequada. Assim, registro brasileiro de síndromes coronarianas agudas^3^ mostrou que cerca de 40% dos pacientes com IAMCSST não receberam nenhuma terapia de reperfusão, e que essas taxas ainda podem ser mais elevadas em determinadas regiões do Brasil.^10^ Vários fatores explicam esse desempenho, como, por exemplo, a demora do paciente em procurar atendimento médico, atraso no diagnóstico e dificuldades nos fluxos regulatórios para encaminhamento desses pacientes para centros terciários de cardiologia.^11^ Por outro lado, a organização de uma rede de telemedicina pode melhorar o desempenho quanto aos dois últimos fatores assim relatados.^12,13^

Com a telemedicina, é possível o envio imediato do ECG para uma central de análises, permitindo que este exame seja interpretado por cardiologista experiente e, assim, otimizando-se a transferência dos pacientes que realmente devem ser encaminhados aos centros terciários. Por exemplo, na presente casuística, o IAMCSST foi suspeitado somente em 15% de todos os ECG enviados pela rede; dessa maneira, otimizou-se o recurso existente para a transferência rápida e o tratamento efetivo deste grupo de pacientes. Previamente à implantação da Rede Supra, o diagnóstico de IAMCSST não se confirmava em muitos pacientes já encaminhados, e isso acaba sobrecarregando o sistema de saúde e dificultando o encaminhamento dos pacientes que realmente precisavam ser transferidos.

Os presentes resultados corroboram os de recente revisão sistemática e metanálise que incluíram 16.960 pacientes e evidenciaram impacto positivo da telemedicina, com redução da taxa de mortalidade intra-hospitalar, RR: 0,63 (IC 95% 0,55 – 0,72), p <0,001, assim como redução no tempo porta-balão, com diferença média de -28 min (IC 95% -35 – -20 min) para o tratamento do IAMCSST.^14^ Nos resultados da presente investigação, é oportuno considerar que a redução da mortalidade intra-hospitalar observada deverá ser interpretada como resultando da combinação do tratamento mais precoce associado à ampliação da angioplastia primária como principal terapia de tratamento desses pacientes.

Os presentes resultados também se alinham aos de outras experiências brasileiras com o uso da telemedicina no atendimento do IAMCSST. Caluza et al.^15^ mostraram uma redução da mortalidade de 26,14% para 7,31%, p = 0,0028 em pronto-atendimentos da região metropolitana de São Paulo após a organização de rede que dispunha de uma central para envio de ECG. Matsuda et al.^16^ também mostraram redução na mortalidade intra-hospitalar muito parecida com a observada na presente investigação (15,00% *vs.* 5,60%) com a utilização da telemedicina para o envio do ECG em outra região da cidade de São Paulo. Marcolino et al.^17^ também relataram uma importante redução na mortalidade intra-hospitalar (12,30% *vs.* 7,10%), p <0,001 com a organização de rede de telemedicina para tratamento do infarto na região de Belo Horizonte. Figueiras Filho et al.^18^ também evidenciaram aumento da porcentagem de pacientes com IAMCSST que receberam terapia de reperfusão (29,10% *vs.* 53,80%), p <0,001 e redução na mortalidade em 30 dias (19,80% *vs.* 5,10%), p <0,001 com a organização de rede para o atendimento do infarto apoiada por recursos de telemedicina na cidade de Salvador.

Além da organização da rede, o estabelecimento de um feedback contínuo entre todas as unidades pertencentes a esta rede é um aspecto fundamental. Conforme mostrado em um estudo prospectivo e multicêntrico alemão, o *feedback* sistemático melhorou os índices de qualidade no atendimento ao IAMCSST, inclusive com redução na mortalidade intra-hospitalar (10,80% vs. 6,80%; p = 0,024).^19^

Aspecto essencial a ser destacado a partir dessa investigação consistiu no uso de plataforma de comunicação digital tão acessível como o WhatsApp^®^, de baixo custo para instalação/manutenção e dispensando treinamento para sua utilização, o que facilita sobremaneira a sua disseminação para outras regiões do país. Vale ressaltar que a qualidade da imagem obtida por esta plataforma em nenhum momento foi impedimento para a análise do ECG; quando houve dificuldades, elas estavam relacionadas à técnica de execução do ECG e não à sua transmissão. Além disso, as mensagens de intercomunicação dos profissionais, assim como as imagens dos ECG, podem ser arquivadas em registro de segurança do sistema.

A Sociedade Brasileira de Cardiologia (SBC) publicou em 2019 uma diretriz sobre telemedicina na qual deixa claro a importância das tecnologias de informação e comunicação para ampliar o acesso aos serviços de saúde no Brasil.^20^ Além disso, nós reforçamos que, após o estabelecimento da Lei Geral de Proteção de Dados em nosso país, deverá ser realizada uma discussão ampliada sobre a segurança da troca de informação médica através do WhatsApp^®^.

### Limitações

Este estudo foi observacional, não randomizado; os dados antes da implantação desta rede de telemedicina foram coletados retrospectivamente por meio de registro de hospitalização, o que pode ter levado à perda de alguns pacientes. Avaliou-se somente a mortalidade intra-hospitalar, e os desfechos após a alta hospitalar não foram analisados. A informação sobre o nível social e educacional dos pacientes incluídos nessa investigação não foi avaliada. Contudo, a organização dessa rede atuou principalmente no atraso do encaminhamento do paciente dentro do sistema de saúde, e não implantou nenhuma intervenção para redução do atraso do paciente em procurar a assistência médica. Assim, consideramos que o nível educacional da população estudada ocasionou pouca repercussão nesses resultados.

## Conclusão

A implantação de rede de telemedicina baseada em plataforma de comunicação acessível como o WhatsApp^®^ para envio e análise do ECG e para organização do fluxo de encaminhamento do paciente com suspeita de IAMCSST para hospital terciário teve imediatos impactos positivos, com aumento da porcentagem de pacientes que receberam terapia de reperfusão e com menor retardo temporal, além de significativa redução da taxa de mortalidade intra-hospitalar.
