# Supplementary material for: Otimização da Terapia de Reperfusão no Infarto Agudo do Miocárdio com Supradesnível do Segmento ST por Meio de Telemedicina Baseada no WhatsApp®
Source: Arq Bras Cardiol. 2022 Jan 11;118(3):556–64. [Article in Portuguese] doi: 10.36660/abc.20201243 (PMC8959040; doi:10.36660/abc.20201243)
Supplement: Translation

# Translation: Reperfusion Therapy Optimization in Acute Myocardial Infarction with ST-Segment Elevation using WhatsApp^®^-Based Telemedicine

**Authors:** Alessandra Batista Teixeira, Leonardo Fiaschi Zancaner, Fernando Fonseca de França Ribeiro, José Paulo Pintyá, André Schmidt, Benedito Carlos Maciel, José Antônio Marin-Neto, Carlos Henrique Miranda

**Affiliations:** 1 Brazil Universidade de São Paulo Faculdade de Medicina de Ribeirão Preto - Divisão de Medicina de Emergência do Departamento de Clínica Médica, Ribeirão Preto, SP - Brazil; 2 Brazil Universidade de São Paulo Faculdade de Medicina de Ribeirão Preto - Centro de Cardiologia, Ribeirão Preto, SP - Brazil

**Keywords:** ST Elevation Myocardial Infarction, Acute Coronary Syndrome, Telemedicine/trends, Reperfusion/therapy

## Abstract

**Background:**

About 40% of patients with ST-segment elevation myocardial infarction (STEMI) in Brazil do not receive reperfusion therapy.

**Objective:**

The use of a telemedicine network based on WhatsApp^®^ could increase the percentage of patients receiving reperfusion therapy.

**Methods:**

A cross-sectional study analyzed outcomes before and after the organization of a telemedicine network to send the electrocardiogram via WhatsApp® of patients suspected of STEMI from 25 municipalities that are members of the Regional Health Department of Ribeirão Preto (DRS−XIII) to a tertiary hospital, which could authorize immediate patient transfer using the same system. The analyzed outcomes included the percentage of patients who received reperfusion therapy and the in-hospital mortality rate. A p value < 0.05 was considered statistically significant.

**Results:**

The study compared 82 patients before (February 1, 2016 to January 31, 2018) with 196 patients after this network implementation (February 1, 2018 to January 31, 2020). After implementing this network, there was a significant increase in the proportion of patients who received reperfusion therapy (60% vs. 92%), relative risk (RR): 1.594 [95% confidence interval (CI) 1.331 – 1.909], p < 0.0001 and decrease in the in-hospital mortality rate (13.4% vs. 5.6%), RR: 0.418 [95%CI 0.189 – 0.927], p = 0.028.

**Conclusion:**

The use of WhatsApp^®^-based telemedicine has led to an increase in the percentage of patients with STEMI who received reperfusion therapy and a decrease in the in-hospital mortality rate.

## Introduction

Cardiovascular diseases are the main cause of mortality worldwide, including in Brazil.^1^ ST-segment elevation Myocardial Infarction (STEMI) is responsible for most of the fatal events of this etiology. According to disclosure by DATASUS, 142,982 patients were hospitalized for acute myocardial infarction in 2018 in Brazil.^1^ In the last decades, the morbidity and mortality of STEMI have greatly reduced, especially with the development of reperfusion therapies (fibrinolytics and primary angioplasty).^2^ However, to obtain this benefit, this coronary event must be recognized early, which is usually based on anamnesis and electrocardiogram (ECG), to allow the organization of the rapid referral of these patients to tertiary centers prepared to offer these types of therapies.

The Brazilian registry of acute coronary syndromes evidenced that only 61.2% of patients with STEMI received reperfusion therapy for treatment (35.9% receiving primary angioplasty and 25.3% receiving fibrinolytic therapy).^3^ Hence, a large percentage of patients in our country, especially those in the public system, still do not receive reperfusion therapy in a timely manner. This directly impacts the survival and functional impairment of these patients’ left ventricle and the consequent heart failure in many STEMI cases.

The objective of this investigation was to evaluate whether the implantation of a telemedicine network to send the ECG of patients with suspected STEMI through a simple digital communication platform (WhatsApp®) for immediate analysis in a tertiary center increased the percentage of patients receiving reperfusion therapy within the initial 12 hours of the STEMI onset. Additionally, we assessed the impact of organizing the immediate release for hospital transfer of a suspected STEMI patient using the same communication resource on the in-hospital mortality due to this coronary event.

## Methods

This before-and-after cross-sectional study compares the percentage of patients who received reperfusion therapy for the treatment of STEMI within a 12-hour period, before and after the implementation of a network (Rede − Supra) for sending patient data and remote analysis of ECGs of patients with this suspected pathology using an accessible digital communication platform (WhatsApp®) to a tertiary cardiology center.

This network included the 25 municipalities included in the Regional Health Department of Ribeirão Preto (DRS − XIII). In this initial phase, it was decided not to include the city of Ribeirão Preto in the data collection, because due to its location, this city already had greater capacity to refer these patients to a tertiary hospital. Figure 1 shows the municipalities that are part of DRS−XIII, as well as their subdivision into three regions, named: Horizonte Verde, Aquífero Guarani, and Vale das Cachoeiras. The receiving center for these ECGs was located in the Coronary Unit of the Emergency Department of Hospital das Clínicas of Ribeirão Preto Medical School at the University of São Paulo (HCFMRP/USP), located in the city of Ribeirão Preto. This is a tertiary referral hospital for the exclusive care and treatment of emergency cases for this entire region.

**Figure 1:** – Geographic map of the municipalities within the Ribeirão Preto Regional Health Department (DRS – XIII); as well as its subdivision into the regions of Horizonte Verde, Aquífero Guarani, and Vale das Cachoeiras.

This study was approved by the Research Ethics Committee of our hospital and followed the recommendations of the Helsinki declaration.

### Implementation of the Supra Network

An initial consultation was carried out with the Regional Council of Medicine, which was in favor of using WhatsApp® to send the ECG between doctors. The project was presented to and approved by the Regional Intergovernmental Commission of the DRS−XIII with the health secretariats of the constituent municipalities.

Two training sessions were conducted with nurses in charge of the emergency care unit in each municipality to demonstrate the importance of early diagnosis and rapid referral of patients with STEMI to a tertiary hospital, since none of these municipalities had primary angioplasty services and fibrinolytic drugs were available in only four of them. A practical training was carried out on how to perform quality ECG, which allows the correct diagnosis and a questionnaire was applied on the resources available in each unit for the care of these patients. After these two activities, the flowchart detailed in figure 2 was presented to the coordinators of these units, containing the guidelines on sending the ECG via WhatsApp® when a STEMI was suspected and the operationalization of the transfer of these patients to the referral hospital (Figure 2).

**Figure 2:** – Flowchart to guide the operationalization of the telemedicine network based on WhatsApp^®^ for sending electrocardiograms (ECG) of suspected ST-segment elevation Myocardial Infarction (STEMI) and organization of the transfer flow of this patient to the tertiary hospital. UE-HC-FMRP - Emergency Unit, Hospital das Clínicas, Ribeirão Preto School of Medicine; CROSS – Center of Regulation for Health Services Offered by the Health Department of the State of São Paulo.

A dedicated cell phone was also made available for this type of communication. The Assistant Cardiologists of the Coronary Unit of the Emergency Department of HCFMRP/USP were responsible for the assessment of these ECGs. Two standard responses were created: confirmed STEMI, immediate transfer to the Emergency Unit was authorized, and unconfirmed STEMI, if necessary was regulated via Health Service Offer Regulation Center (CROSS – *Central de Regulação de Ofertas e Serviços de Saúde*). On February 1, 2018, the dedicated cell phone number was made available to all these municipalities.

### Data collection

Retrospective data prior to the implementation of this network were acquired by reviewing the medical records of patients admitted to our hospital with a primary diagnosis of STEMI, identified through the following international code of diseases (ICD−10): I21.0 (acute transmural myocardial infarction of anterior wall), I21.1 (acute transmural myocardial infarction of inferior wall), I21.2 (acute transmural myocardial infarction of other sites), I21.3 (acute transmural myocardial infarction of unspecified site), I22.0 (subsequent myocardial infarction of anterior wall), I22.1 (subsequent myocardial infarction of inferior wall), I22.8 (subsequent myocardial infarction of other sites), I22.9 (subsequent myocardial infarction of unspecified site) The only analyzed cases were those referred by the 25 municipalities that comprise the DRS XIII in the two-year period before the implementation of this telemedicine network. The definition for the diagnosis of STEMI followed the criteria of the Fourth Universal Definition of Myocardial Infarction.^4^

After the implementation of the Rede Supra, the data were prospectively collected by a nurse from the Coronary Unit who weekly reviewed the messages and ECGs sent to this center via WhatsApp® and subsequently checked the information necessary for this study in all patients’ records during the two years following the implementation of this network.

The primary assessed outcome was the percentage of patients who received some type of reperfusion therapy (fibrinolytics, primary angioplasty or spontaneous reperfusion) within 12 hours of the onset of chest pain symptoms. The secondary assessed outcomes were the in-hospital mortality rate, as well as the time between chest pain onset and the reperfusion therapy start. For patients with spontaneous reperfusion or for those who did not receive any type of reperfusion therapy, the time between pain onset and hospital admission was considered.

### Statistical analysis

To determine the sample size using the chi-square test, it was assumed that 50% of patients from this region received reperfusion therapy before this network was implemented, based on recent historical data from our institution. In addition, it was assumed that this percentage would rise to 80% after the implementation of this network, detecting this difference with power of 80% and a significance level of 5%. Thus, the study would need to include at least 50 patients (before) and 50 patients (after) to test this hypothesis.

The Shapiro-Wilk test was used to assess the type of distribution of quantitative variables. Quantitative variables with normal distribution were expressed as mean ± standard deviation and the other variables as median and interquartile range (IQR). To compare the two quantitative variables with normal distribution, the unpaired Student’s *t* test was used. The Mann-Whitney test was employed for those with another type of distribution. Qualitative variables were expressed as frequencies and percentages. To compare between two or more qualitative variables, the chi-square test was used. To evaluate the association between two variables, the relative risk (RR) was calculated, as well as its 95% confidence interval (95% CI). A two-tailed p-value <0.05 was considered statistically significant. The statistical analysis and construction of the graphs were performed using the GraphPad Prism statistical software, version 7.00 (CA, USA).

## Results

From February 1, 2018 through January 31, 2020, the ECG of 1847 patients were sent through this network and evaluated. Of these ECGs, 280 (15%) patients were suspected to have STEMI, which was confirmed in 196 (11%) after clinical evaluation and after the ECG was repeated in the hospital setting. The time between receiving the ECG through WhatsApp® and sending the response was less than 10 minutes in the vast majority of cases. The other characteristics of patients whose ECGs were analyzed through this telemedicine network are shown in Table 1.

**Table 1:** – Characterization of patients whose electrocardiograms were sent through the Rede Supra via WhatsApp® from February 1, 2018 through January 31, 2020

Characteristics		n = 1847
ECG with suspected ST-elevation, n (%)		280(15)
ECG with confirmed ST-elevation, n (%)		196(11)
**Response time, n (%)**		
< 10 min		1651(89)
10 – 30 min		125(07)
30 – 60 min		36(02)
> 60 min		35(02)
**Age group, n (%)**		
< 40 years		268(15)
≤40 – <50 years		261(14)
≤50 – <60 years		379(21)
≤60 – <70 years		395(21)
≥70 years		416(23)
Not informed		128(06)
**Gender, n (%)**		
Male		1033(56)
Female		541(29)
Not informed		273(15)
**Origin, n (%)**		
Horizonte Verde		645(35)
Aquífero Guarani		596(32)
Not informed		332(18)
Vale das Cachoeiras		274(15)

*ECG: electrocardiogram; min: minutes.*

The demographic and clinical characteristics of patients diagnosed with STEMI treated at our service before and after the implantation of this network are shown in Table 2. There was no difference in relation to age. Despite the predominance of males in the two analyzed periods, the proportion of females clearly increased in the second period. No difference was observed in relation to the personal history of these patients or in relation to the affected left ventricular walls.

**Table 2:** – Characteristics of patients at hospital admission before and after the implementation of the Rede Supra

Characteristics		Before (n = 82 patients)	After (n = 196 patients)	p-value
**Demographics**				
Age, mean ± SD		60 ± 11	61 ± 12	0.676
Male gender, n (%)		65(79)	123(63)	0.007
**Personal history, risk factors**				
Hypertension, n (%)		46(56)	112(57)	0.873
Diabetes, n (%)		21(26)	57(29)	0.661
Dyslipidemia, n (%)		21(26)	42(21)	0.448
Smoker, n (%)		41(50)	92(47)	0.641
Prior AMI, n (%)		6(07)	21(11)	0.383
Prior angioplasty, n (%)		2(02)	15(08)	0.098
Prior CABG, n (%)		0(00)	00(00)	1.000
**Physical examination on admission**				
SBP (mmHg), mean ± SD		135 ± 27	122 ± 27	0.0009
DBP (mmHg), mean ± SD		74 ± 15	82 ± 17	<0.0001
HR (beats/min), mean ± SD		80 ± 20	85 ± 18	0.072
SBP <90 mmHg, n (%)		7(09)	8(04)	0.134
**Affected LV wall, n (%)**				0.155
Anterior		27(33)	90(46)	
Inferior		53(65)	96(49)	
Other		2(02)	10(05)	
Troponin (μg/L), median (IQR)		9.87(3.28 – 20.09)	13.50(5.00 – 30.00)	0.058
**Origin, n (%)**				0.043
Vale das Cachoeiras		18(22)	68(35)	
Horizonte Verde		29(35)	62(32)	
Aquífero Guarani		35(43)	66(34)	
Hospital ength of stay (days), median (IQR)		5(4 – 9)	5(4 – 9)	0.845

*SD: standard deviation; AMI: acute myocardial infarction; CABG: Coronary Artery Bypass Grafting ; SBP: systolic blood pressure; DBP: diastolic blood pressure; HR: heart rate; LV: left ventricle; IQR: interquartile range.*

After the implantation of the Rede Supra, there was a statistically significant increase in the proportion of patients who received reperfusion therapy for acute treatment of STEMI, 49/82 (60.00%) *vs*. 180/196 (92.00%), RR: 1.594 (95% CI 1.331 – 1.909), p <0.0001, as depicted in Figure 3A. In terms of the type of reperfusion therapy used in the treatment, a statistically significant increase was observed in the proportion of patients treated with primary angioplasty. The proportion of patients who received a fibrinolytic agent or who had spontaneous reperfusion was similar before and after, as shown in Figure 3B.

**Figure 3:** – Bar graph showing the percentage of patients who received some type of reperfusion therapy before and after the implementation of the Rede Supra (A); as well as the types of reperfusion therapy received for the treatment of acute ST-segment elevation Myocardial Infarction (STEMI) (B). Spontaneous - coronary recanalization was spontaneous without fibrinolytics or primary angioplasty.

Moreover, the in-hospital mortality rate of patients with STEMI in our service was significantly reduced, 11/82 (13.40%) *vs*. 11/196 (5.60%), RR: 0.418 (95% CI 0.189 – 0.927), p = 0.028, as depicted in Figure 4A. These values indicate a number needed to treat (NNT) of 12 corresponding to a reduction in mortality. The time between chest pain onset and the reperfusion therapy start was significantly reduced from 9 h [IQR 6 - 19] in the previous period, to 4 h [IQR 3 - 6] in the period after the network implementation, p <0.0001.

**Figure 4:** – Bar graph illustrating in-hospital mortality (A) and boxplot showing the time between symptom onset and the start of reperfusion therapy (B) of patients with confirmed diagnosis of ST-segment elevation Myocardial Infarction (STEMI) before and after the implementation of the Rede Supra.

## Discussion

This investigation found that the implementation of a telemedicine network using an accessible digital communication platform (WhatsApp®) to send the ECG for specialized analysis and the organization of the influx of patients with suspected STEMI to a tertiary referral hospital for treatment of this pathology, was associated with a significant increase in the proportion of patients who received reperfusion therapy, a reduction in the time to implement this therapy, and a significant reduction in the in-hospital mortality rate.

Scientific evidence has demonstrated that the rapid initiation of reperfusion therapy to treat STEMI significantly reduces complications, mainly by minimizing myocardial damage.^5,6^ Based on this observation, the organization of regional care networks for patients with STEMI is recommended in an attempt to expedite their treatment.^7-9^

In developing countries, such as Brazil, a significant percentage of patients with STEMI still do not receive reperfusion therapies within an adequate time window. Thus, a Brazilian report on acute coronary syndrome^3^ showed that about 40% of patients with STEMI did not receive any reperfusion therapy, and these rates may be even higher in certain regions of Brazil.^10^ Several factors explain this occurrence, such as the patient’s delay in seeking medical care, underdiagnosis and difficulties in the regulatory influxes for referring these patients to tertiary cardiology centers.^11^ On the other hand, the organization of a telemedicine network can improve performance for these two latter factors.^12,13^

Telemedicine facilitates the prompt sending of the ECG to an analysis center, allowing this test to be interpreted by an experienced cardiologist and, thus, optimizing the transfer of patients who really should be referred to tertiary centers. For example, in this case series, STEMI was suspected in only 15% of all ECGs sent through the network, thus optimizing the existing resources for rapid transfer and effective treatment of this group of patients. Prior to the implementation of the Rede Supra, the diagnosis of STEMI was not confirmed in many referred patients, resulting in an overload of the health system and making it difficult to refer patients who really need to be transferred.

The present results corroborate those of a recent systematic review and meta-analysis that included 16,960 patients and evidenced a positive impact of telemedicine, with a reduction in the in-hospital mortality rate, (RR: 0.63; 95% CI 0.55 – 0.72, p <0.001), as well as a reduction in door-to-balloon time, with an average difference of -28 min (95% CI -35 – -20 min) for the treatment of STEMI.^14^ According to the results of the present investigation, the reduction observed for in-hospital mortality should be interpreted as resulting from the combination of the earlier treatment associated with the expansion of primary angioplasty as the main treatment for these patients.

The present results are also in line with those of other Brazilian authors regarding the use of telemedicine in the care of STEMI. Caluza et al.^15^ showed a reduction in mortality from 26.14% to 7.31%, p = 0.0028 in emergency rooms in the metropolitan region of São Paulo after the implementation of a network with a central contact to send ECGs. Matsuda et al.^16^ also detected a reduction in in-hospital mortality very similar to that observed in the present investigation (15.00% vs. 5.60%) with the use of telemedicine to send the ECG in another region of the city of São Paulo. Marcolino et al.^17^ reported an important reduction in in-hospital mortality (12.30% vs. 7.10%, p <0.001) with the implementation of a telemedicine network for the treatment of infarction in the Belo Horizonte region. Figueiras Filho et al.^18^ also observed an increase in the percentage of patients with STEMI who received reperfusion therapy (29.10% vs. 53.80%, p <0.001), and a reduction in 30-day mortality (19.80% vs. 5.10%, p <0.001) with the network implementation for infarction care supported by telemedicine resources in the city of Salvador.

In addition to the implementation of the network, the establishment of a continuous feedback between all units belonging to this network is a fundamental aspect. A prospective and multicenter German study reported that systematic feedback improved quality indexes in STEMI care, including a reduction of in-hospital mortality (10.80% vs. 6.80%; p = 0.024).^19^

An essential aspect to be highlighted from this investigation was the use of an accessible digital communication platform, such as WhatsApp®, with low-cost installation/maintenance and that does not require training for its use, which greatly facilitates its dissemination to other regions of the country. It is worth mentioning that the image quality obtained by this platform never hindered the analysis of the ECG; when there were difficulties, they were related to the ECG performance technique and not to the ECG transmission. In addition, the intercommunication messages between professionals, as well as the ECG images, can be maintained in the system’s security record.

The Brazilian Society of Cardiology (SBC) published in 2019 a guideline on telemedicine, in which it makes clear the importance of information and communication technologies to expand access to health services in Brazil.^20^ Furthermore, we reinforce that after the establishment of the General Data Protection Act in our country, an expanded discussion on the security of medical information exchange through WhatsApp® should be conducted.

### Limitations

This was an observational, non-randomized study, and the data before the implementation of this telemedicine network were collected retrospectively through hospitalization records, which may have led to the loss of data from some patients. Only in-hospital mortality was assessed, and outcomes after hospital discharge were not analyzed. Information on the social and educational level of the patients included in this investigation was not evaluated. However, the establishment of this network mainly minimized the delaying in referring the patient within the health system and did not implement any intervention to reduce the patient’s delay in seeking medical assistance, so the educational level of the studied population had little repercussion on these results.

## Conclusion

The implementation of a telemedicine network based on an accessible communication platform, such as WhatsApp®, for sending and analyzing ECGs, and for organizing the referral influx of patients with suspected STEMI to a tertiary hospital had immediate positive impacts, with an increase in the percentage of patients who received reperfusion therapy and with less delay, in addition to a significant reduction in the in-hospital mortality rate.
